# Reliability of wing morphometrics for species identification of human-biting black flies (Diptera: Simuliidae) in Thailand

**DOI:** 10.1186/s13071-024-06597-8

**Published:** 2024-12-18

**Authors:** Kittipat Aupalee, Wichai Srisuka, Kwankamol Limsopatham, Sangob Sanit, Hiroyuki Takaoka, Atiporn Saeung

**Affiliations:** 1 Parasitology and Entomology Research Cluster (PERC), Department of Parasitology, Faculty of Medicine, Chiang Mai University, Chiang Mai, 50200 Thailand; 2 Entomology Section, Queen Sirikit Botanic Garden, Mae Rim, Chiang Mai, 50180 Thailand; 3 Tropical Infectious Diseases Research and Education Centre (TIDREC), Higher Institution Centre of Excellence (HICoE), Universiti Malaya, 50603 Kuala Lumpur, Malaysia

**Keywords:** Morphometric analysis, DNA barcodes, Species delimitation, Hematophagous insect, Medical entomology, *Simulium*

## Abstract

**Background:**

Fast and reliable species identification of black flies is essential for research proposes and effective vector control. Besides traditional identification based on morphology, which is usually supplemented with molecular methods, geometric morphometrics (GM) has emerged as a promising tool for identification. Despite its potential, no specific GM techniques have been established for the identification of black fly species.

**Methods:**

Adult female black flies collected using human bait, as well as those reared from pupae, were used in this study. Here, landmark-based GM analysis of wings was assessed for the first time to identify human-biting black fly species in Thailand, comparing this approach with the standard morphological identification method and DNA barcoding based on the mitochondrial cytochrome *c* oxidase subunit I (*COI*) gene. To explore genetic relationships between species, maximum likelihood (ML) and neighbor-joining (NJ) phylogenetic trees were built. Additionally, three different methods of species delimitation, i.e., assemble species by automatic partitioning (ASAP), generalized mixed yule coalescent (GMYC), and single Poisson tree processes (PTP), were utilized to identify the morphologically defined species. The effectiveness of a *COI* barcode in identifying black fly species was further examined through the best match (BM) and best close match (BCM) methods.

**Results:**

Seven black fly species, namely *Simulium tenebrosum* Takaoka, Srisuka & Saeung, 2018 (complex), *S. doipuiense* Takaoka & Choochote, 2005 (complex), *S. nigrogilvum* Summers, 1911, *S. nodosum* Puri, 1933, *S. asakoae* Takaoka & Davies, 1995, *S. chamlongi* Takaoka & Suzuki, 1984, and *S. umphangense* Takaoka, Srisuka & Saeung, 2017 were morphologically identified. Compared with the standard method, the GM analysis based on wing shape showed high success in separating species, achieving an overall accuracy rate of 88.54%. On the other hand, DNA barcoding surpassed wing GM for species identification with a correct identification rate of 98.57%. Species delimitation analyses confirmed the validity of most nominal species, with an exception for *S. tenebrosum* complex and *S. doipuiense* complex, being delimited as a single species. Moreover, the analyses unveiled hidden diversity within *S. asakoae*, indicating the possible existence of up to four putative species.

**Conclusions:**

This study highlights the potential of wing GM as a promising and reliable complementary tool for species identification of human-biting black flies in Thailand.

**Graphical Abstract:**

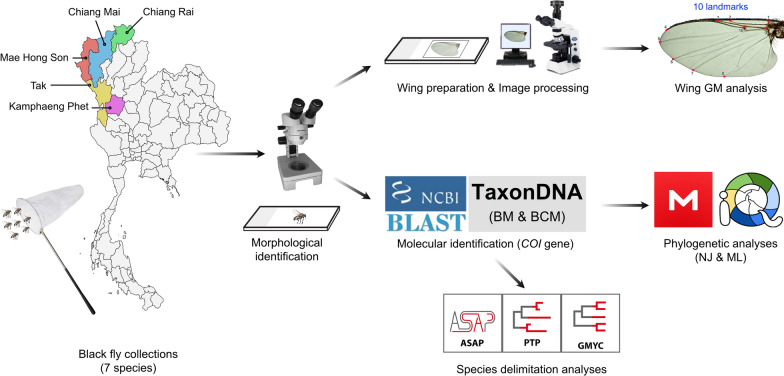

**Supplementary Information:**

The online version contains supplementary material available at 10.1186/s13071-024-06597-8.

## Background

Black flies are small, dark, humpbacked flies that belong to the family Simuliidae, with more than 2400 species formally recorded worldwide [1]. The female flies usually feed on the blood of birds and mammals, including humans, which can cause irritation, pain, swelling, and redness at the bite site due to an immunoglobulin (Ig)E-mediated reaction to salivary gland proteins [2–9]. During the biting, some species could transmit various pathogens to both animals and humans, especially the filarial worm *Onchocerca volvulus*, the causative agent of human onchocerciasis or river blindness [9, 10]. This disease is the second leading cause of infectious blindness globally and is one of the 21 neglected tropical diseases (NTDs) that the World Health Organization (WHO) has targeted for elimination by 2030 [11]. In Thailand, a total of 146 species of black flies have been officially documented, with seven species (*Simulium asakoae*, *S. nigrogilvum*, *S. tenebrosum* complex, *S. doipuiense* complex, *S. umphangense*, *S. chamlongi*, and *S. nodosum*) considered to be human biters [12–15]. Most recently, host blood meal analysis of adult flies based on mitochondrial cytochrome *b* (*cytb*) gene revealed that two other species, *S. chumpornense* Takaoka & Kuvangkadilok, 2000 and *S. striatum* species-group fed on human blood [16]. However, no human infectious diseases transmitted by these flies have been reported so far in the country [17]. Notably, three human-biting species, namely *S. asakoae*, *S. nigrogilvum* and *S. nodosum*, have been incriminated as natural vectors of various species of filarial or nonfilarial worms, including the genus *Onchocerca* [17–22]. Furthermore, *S. chumpornense*, *S. khelangense* Takaoka, Srisuka & Saeung, 2022, and *S. asakoae* were recently identified as natural vectors of avian blood protozoa of the genera *Leucocytozoon* and *Trypanosoma* [23–26]. Considering the critical role of black flies as vectors for transmitting diverse pathogens, rapid and accurate species identification is crucial for comprehending disease epidemiology and developing effective vector control measures [27].

Traditionally, black fly species identification relies on meticulous examination of morphological characteristics of a large series of larvae, pupae, and adults (males and females), sometimes even requiring dissection [28]. Due to the small size and morphological uniformity of this insect, morphological classification is extremely difficult and often requires trained experts, proving impractical or even impossible for specimens that are damaged or closely related species [28, 29]. To overcome these limitations, other methods (cytogenetics and DNA barcoding) have been developed and used together to assist black fly species identification and confirmation [29–32]. Currently, DNA barcoding based on *COI* gene sequences plays a significant role in black fly species identification, enabling researchers to distinguish species rapidly and accurately. This method is not only used as an effective identification tool, but can also reveal hidden diversity within nominal species [28, 33–39]. Nevertheless, molecular approaches are relatively expensive, sophisticated, and need to be conducted in well-equipped laboratories [40, 41]. A faster, cost-effective, easy to use, and reliable method for accurately identifying species is necessary.

Over the past decade, geometric morphometrics (GM) analysis has emerged as a potential game changer, being used as an effective complementary tool for the species identification of various insect groups, particularly those with medical and veterinary importance [41–50]. This approach has also proved to be a valuable tool for detecting sexual dimorphism, recognition of cryptic diversity, and studying evolution [48, 51–55]. Presently, the landmark-based GM method of insect wings is the most popular and is a powerful tool used for supplementing and enhancing morphological identification [56, 57]. This method analyzes wing size and shape based on the positions of anatomical landmarks (also known as true landmarks) and the distances between them, which includes both size and shape data [56, 57]. While GM analysis has been employed successfully for distinguishing species of several insect groups, its application for black fly identification remains unexplored. To the best of our knowledge, there is only one study that applied this technique for interpreting evolutionary transitions in the black fly wings [51].

In the present study, landmark-based GM analysis of wings was evaluated, for the first time, for the identification of seven human-biting black fly species of Thailand. For comparison, DNA barcoding based on the *COI* gene was used to distinguish the species and the effectiveness of this gene for species identification was also tested using the best match (BM) and best close match (BCM) methods. Additionally, three species delimitation methods [assemble species by automatic partitioning (ASAP), generalized mixed yule coalescent (GMYC), and single Poisson tree processes (PTP)] were employed to identify the recognized morphospecies.

## Methods

### Black fly samples and morphological identification

Most adult female flies were caught with a sweep net while flying around a human bait, while others were reared from pupae collected with fine forceps from available substrates in streams. The wild-caught females were promptly euthanized by submerging them in a 15 ml centrifuge tube containing 80% alcohol. Meanwhile, matured pupae were kept individually in a 15 ml centrifuge tube with minimal water at the bottom until adulthood. The emerged adult females were kept alive in the same tube for at least 24 h to ensure the hardening and coloration of their exoskeleton [15]. All specimens were preserved in 80% ethanol and stored in a freezer (−20 °C) until further analysis. Species identification of the adult flies was based on the examination of their morphological characteristics under a stereomicroscope (Olympus SZ51, Japan) using the standard keys for the black flies of Thailand [58]. In total, 253 adult female black flies (seven species or complex) belonging to the subgenera *Gomphostilbia* [one species designated here as “*S. asakoae*” although our specimens probably include not only *S. asakoae* but also several other species of the *S. asakoae* species-group, which are morphologically indistinguishable from one another (*n* = 50)] and *Simulium* s. str. [six species including *S. chamlongi* (*n* = 30), *S. doipuiense* complex (*n* = 39), *S. nigrogilvum* (*n* = 39), *S. nodosum* (*n* = 31), *S. tenebrosum* complex (*n* = 31), and *S. umphangense* (*n* = 33)] of the genus *Simulium* Latreille s. l. were utilized in this study as outlined in Table 1.

**Table 1:** Details of adult female black flies used in this study

Species	Sampling site		Coordinate (latitude/longitude)	Elevation (m)	Date of collection	*n*	Total
*S. asakoae*	Ban Pang Dang, Doi Saket, Chiang Mai		19°03′35.0″ N/99°21′52.9″ E	932	28 August 2023	25	50
	Ban Pang Bong, Doi Saket, Chiang Mai		18°49′04.3″ N/99°20′06.2″ E	981	28 August 2023	25	
*S. chamlongi*	A-Frame, Doi Pha Hom Pok, Chiang Mai		20°02′12.9″ N/99°09′55.8″ E	1529	29 November 2012	21^a^	30
	Mae Kam Pong Waterfall, Mae On, Chiang Mai		18°51′48.6″ N/99°21′22.9″ E	1076	1 June 2015	4^a^	
	Pangkhon, Mueang, Chiang Rai		19°54′17.6″ N/99°35′54.0″ E	1386	29 October 2016	5^a^	
*S. doipuiense* complex	Mae Klong Kee, Umphang, Tak		16°13′33.0″ N/98°58′46.8″ E	1264	24 June 2021	2	39
	Mae Klong Kee Unit, Umphang, Tak		16°14′45.8″ N/98°59′52.1″ E	1188	17 August 2021	12	
			16°13′30.9″ N/98°58′47.3″ E	1237	18 August 2021	16	
	Pa Deuk Dum Bun, Umphang, Tak		16°14′38.0″ N/98°59′55.6″ E	1263	19 August 2021	9	
*S. nigrogilvum*	Mae Klong Kee, Umphang, Tak		16°13′29.1″ N/98°58′44.9″ E	1279	24 June 2021	12	39
	Mae Klong Kee Unit, Umphang, Tak		16°14′45.8″ N/98°59′52.1″ E	1188	17 August 2021	19	
			16°13′30.9″ N/98°58′47.3″ E	1237	18 August 2021	8	
*S. nodosum*	Tham Pla, Mueang, Mae Hong Son		19°30′09.2″ N/98°00′22.9″ E	385	24 October 2014	20	31
	Tao Dam Waterfall, Khlonglan, Kamphaeng Phet		16°18′04.6″ N/99°06′44.3″ E	539	20 December 2016	11^a^	
*S. tenebrosum* complex		Doi Inthanon, Chom Thong, Chiang Mai	18°35′12.8″ N/98°29′14.2″ E	2534	19 December 2018	31	31
*S. umphangense*		Ban Lek, Doi Pha Hom Pok, Chiang Mai	20°04′30.9″ N/99°11′07.4″ E	1468	29 May 2010	1	33
		Chong Yen, Mae Wong, Kamphaeng Phet	16°06′02.3″ N/99°06′29.0″ E	1276	23 March 2016	3	
					19 January 2017	13	
					4 November 2018	3	
		Mae Klong Kee Unit, Umphang, Tak	16°13′34.8″ N/98°58′46.1″ E	1272	1 March 2013	6	
		Upstream Mae Klong Yai, Umphang, Tak	16°18′00.5″ N/99°01′22.6″ E	1095	22 March 2016	1	
		Pa Deuk Dum Bun, Umphang, Tak	16°14′38.0″ N/98°59′55.6″ E	1263	5 March 2020	3	
		Mae Klong Kee, Umphang, Tak	16°13′33.0″ N/98°58′46.8″ E	1264	24 June 2021	3	

^a^Adult female reared from pupa

### Wing preparation, image processing, and landmark digitization

After morphological identification of the species, at least 30 specimens of each black fly species with intact wings were selected for geometric morphometric analysis (Table 1). A subset of these fly samples (ten specimens/species) was also randomly chosen for molecular analysis.

**Fig. 1:**
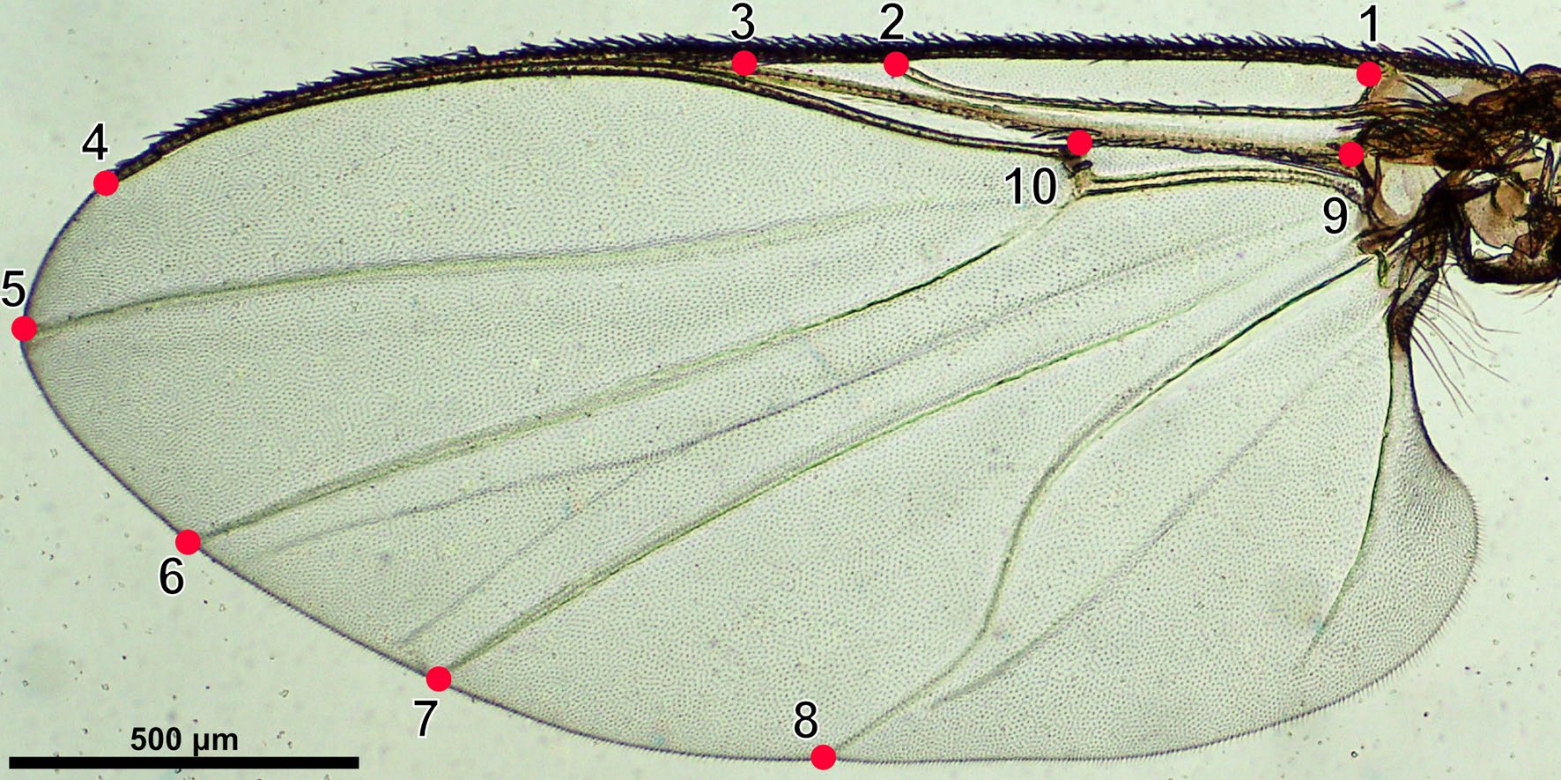
A representative image of black fly wing showing ten landmarks used in geometric morphometric analysis

To prepare a semi-permanent slide, the right wing of each individual was first removed from the thorax using an insect needle under a stereomicroscope (Olympus SZ51, Japan). Each wing was then transferred to a new slide, placed on a drop of 80% alcohol, and covered with a coverslip. To prevent leakage and evaporation of the alcohol while observing and photographing the specimens, air-drying nail polish (Revlon, Indonesia) was applied to seal the edges of a coverslip. Digital images of each wing were captured using a DP27 digital camera attached to a 4× magnification light microscope (Olympus CX41, Japan). All images were annotated with a 500 μm reference scale bar. A total of ten landmarks (Fig. 1) slightly modified from [51] were digitized on each wing.

### Repeatability

To assess the accuracy of digitizing landmarks, a repeatability test for the shape was performed. Ten wing images of each fly species were randomly selected and digitized twice by the same user for intra-user repeatability. The measurement error, expressed as the repeatability index (*R*), was calculated by comparing two sets of digitized images using the Procrustes analysis of variance (ANOVA) method [59].

### Allometry

As previously reported, wing sizes could influence wing shape variation (allometry) [60, 61]. This correlation was examined before conducting the wing shape analysis. The coefficient of determination (*R*^2^), obtained from analyzing the linear regression between the wing centroid size (CS) and the first principal component (PC) of wing shape, was used to assess the extent of the influence.

### Wing size and shape analysis

To estimate the global wing size of each species, we employed the centroid size (CS), which was derived from the distances between the centroid point of each configuration and each landmark [62]. The variation of wing CS among *Simulium* species was illustrated using a violin plot. Statistically significant differences in wing CS between species were assessed using a one-way ANOVA (1000 iterations) with Bonferroni adjustment for significance testing at a *p*-value < 0.05.

For the wing shape analysis, shape variables were derived through a Procrustes superimposition of landmark configurations using the generalized Procrustes analysis (GPA) [63, 64]. Subsequently, partial wrap scores generated from principal component analysis were used as final shape variables for subsequent analyses. To explore species separation, discriminant analysis (DA) was performed using the final shape variables as an input and was illustrated as a factor map. The Mahalanobis distance, obtained from DA analysis, was calculated to estimate shape divergence between species. Wing shape differences among species based on the Mahalanobis distances were analyzed using a nonparametric permutation test (1000 iterations) with a Bonferroni adjustment for significance testing at a *p*-value < 0.05. Additionally, a hierarchical clustering tree (UPGMA algorithm) based on Mahalanobis distances was constructed to assess the relationships of wing shape among species [65].

### Validated classification

A cross-validated classification (jackknife classification) was performed to test the accuracy of wing size and shape for correct species identification [66]. Each individual was successively excluded from the total sample and then allocated to the most probable group for size and the nearest group for shape using the maximum likelihood method [67] and Mahalanobis distance [68], respectively.

### Morphometric software

The geometric morphometrics analysis, including landmark digitization, repeatability test, allometric examination, wing size and shape analyses, and a cross-validated classification test were performed using the online application XY Online Morphometrics (XYOM) version 2 [69]. The software is freely accessible at https://xyom.io/, accessed on 10 March 2024. In addition, the results generated from the XYOM were further beautified in RStudio [70].

### Molecular identification based on DNA barcoding

For supplementing the morphological species identification, DNA barcoding using the *COI* gene was performed on the randomly selected specimens of each black fly species (ten specimens/species). Total DNA was extracted from the thorax of individual flies using the TIANamp Genomic DNA Kit (TIANGEN Biotech, Beijing, China), according to the manufacturer’s protocol. A DNA fragment of the mitochondrial *COI* gene (658 bp in length) was amplified using the universal primers: LCO1490 (5′-GGT CAA CAA ATC ATA AAG ATA TTG G-3′) and HCO2198 (5′-TAA ACT TCA GGG TGA CCA AAA AAT CA-3′) [71]. Each PCR reaction (20 μl total volume) was composed of 2 μl of DNA template, 1 U of Taq DNA polymerase, 3 mM MgCl_2_, 0.2 mM dNTPs, and 0.2 μM of each primer. The PCR cycling conditions included: an initial denaturation at 94 °C for 2 min followed by 40 cycles at 94 °C for 30 s, 50 °C for 45 s, and 72 °C for 45 s, with a final extension at 72 °C for 5 min. Subsequently, the PCR products were checked by agarose gel electrophoresis (1.5%), visualized by non-toxic Ultrapower (BioTeke, Beijing, China) dye. All PCR samples with a band of the expected size (658 bp) were sent to First Base Laboratories Sdn Bhd (Malaysia) for purifying and sequencing using the BigDye Terminator v.3.1 cycle sequencing kit on an ABI 3730XL Genetic Analyzer (Applied Biosystems Inc., Foster City, CA, USA). To generate a consensus sequence of each specimen, both forward and reverse sequences were assembled and edited manually in Geneious Prime 2024.0.5 [72]. Calculation of intra- and interspecific genetic distances based on the Kimura two-parameter (K2P) model [73] was conducted in MEGA 11 [74, 75]. The species identities of each black fly specimen were determined by comparing the newly generated sequences with previously published sequences deposited in the GenBank database, using the basic local alignment search tool (BLAST), available at http://blast.ncbi.nlm.nih.gov/Blast.cgi. Success rates of the DNA barcoding in species identification were evaluated based on the BM and BCM methods using the TaxonDNA [76]. All *COI* sequences obtained in the present study were deposited in the GenBank database under the following accession numbers: PP422429–PP422472.

### Multiple sequence alignment and phylogenetic analysis

All sequences obtained in this study and others fetched from GenBank database were aligned with MUSCLE 3.7 [77], performed in MEGA 11 [74, 75]. Subsequently, the *COI* alignment was used to infer the genetic relationships among seven human-biting black fly species based on the neighbor-joining (NJ) and maximum likelihood (ML) methods. The NJ tree was constructed in MEGA 11 based on the K2P model with 1000 bootstrap iterations [74, 75, 78], while the ML tree was reconstructed using IQ-TREE version 2.3.1 [79] with 10,000 ultrafast bootstrap iterations [80]. The best-fitting substitution model for the ML method, which was K3Pu + F + I + R2, was selected using ModelFinder based on Bayesian Information Criterion (BIC) [81]. The final tree, visualized by FigTree version 1.4.4 (http://tree.bio.ed.ac.uk/software/figtree/), was rooted using the *COI* sequence of *S. khongchiamense* Takaoka, Srisuka & Saeung, 2023, a member of the subgenus *Asiosimulium*.

### Species delimitation analysis

Three different methods of DNA sequence-based species delimitation, including ASAP, GMYC, and single PTP were performed to estimate the number of molecular operational taxonomic units (MOTUs). ASAP, a distance-based method, was conducted online using the ASAP webserver, available at https://bioinfo.mnhn.fr/abi/ public/asap/ , with default parameters [82]. The PTP analysis was run through mPTP webserver (https://mptp.h-its.org/#/tree) using the maximum likelihood implementation with a single Poisson distribution [83]. The ML tree generated from IQ-TREE was used as the input tree in the PTP analysis with a default *p*-value of 0.001. For the GMYC analysis, an ultrametric tree was generated with BEAUti2 software under the Yule process speciation model and the relaxed clock log-normal model [84, 85]. The best fitting substitution model (TrN + I + G) based on the BIC was determined using jModelTest 2.1.7 [86]. The MCMC chain was run in BEAST v2.6.7 for 20 million iterations with sampling frequency of 1000 iterations each. As previously recommended [87], the output file was checked using Tracer v1.7 software to ensure that all the effective sample size (ESS) values were greater than 200. To infer a maximum clade credibility tree from the set of posterior trees, the output tree was subjected to TreeAnnotator v2.6.7 with a burn-in of 20% [85]. The resulting tree was then analyzed under the single-threshold operation using the R package “splits” on the R platform [70, 88].

## Results

### Repeatability

The repeatability test for the wing shape revealed a very low measurement error score (1%) and a high repeatability score (99%), suggesting a high level of accuracy in landmark placement on the tested wing image set.

### Allometric effect

**Fig. 2:**
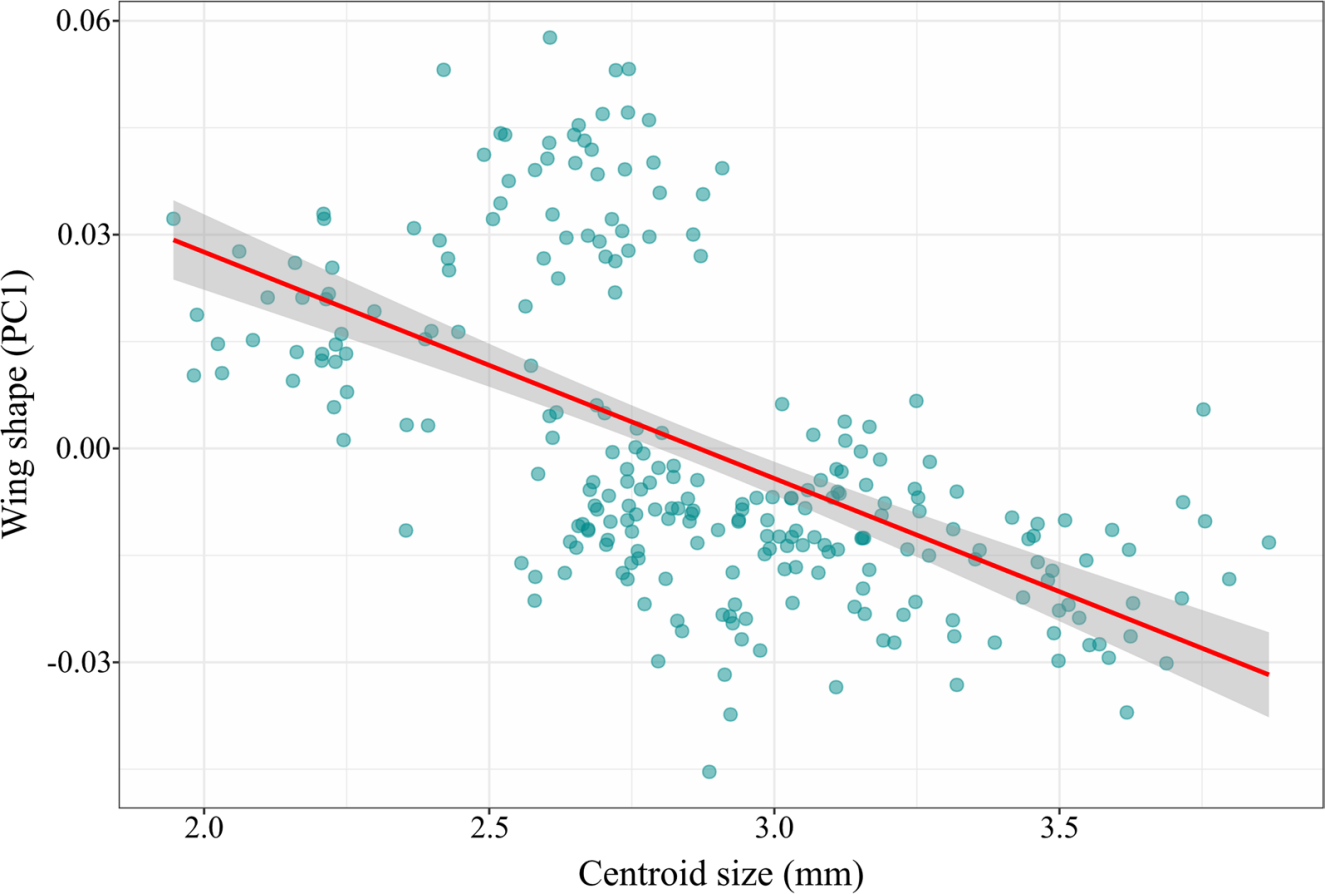
Scatter plot showing the allometric relationship between the wing shape (the first principal component, PC1) and wing size (centroid size) of seven black fly species. The red line indicates the linear regression prediction with 95% confidence intervals (shaded areas), while the sapphire dots represent individual samples

Assessment of the allometric effect revealed an apparent negative relationship between wing size and wing shape (*R*^2^ = 33.7%) with statistical significance (*p* < 0.05) (Fig. 2). This indicated that wing size variation influences wing shape divergence between species.

### Wing size variation

**Fig. 3:**
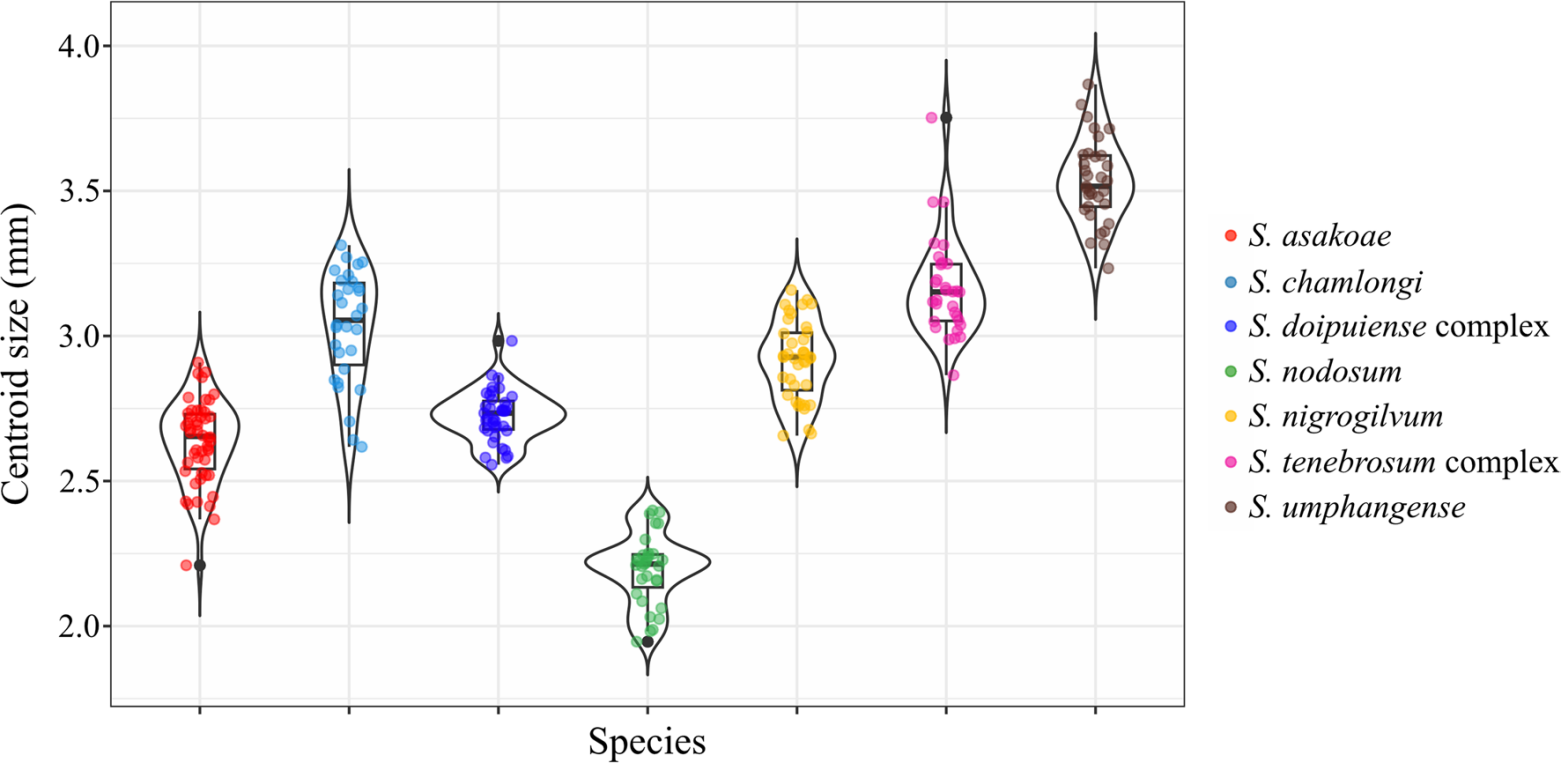
Violin plot overlaid with a box plot showing the distribution of wing centroid sizes (CS) in millimeters (mm) of seven black fly species

The variation in wing size (CS) among seven human-biting black fly species is depicted in Fig. 3. *Simulium umphangense* displayed the largest wing size of 3.53 ± 0.15 mm (mean ± S.D.), while *S. nodosum* exhibited the smallest wing size at 2.19 ± 0.12 mm (Table 2). The comparisons of wing size based on a nonparametric permutation test (1000 iterations) with Bonferroni adjustment revealed significant differences (*p* < 0.05) between almost all species pairs, with the exception of *S. asakoae* versus *S. doipuiense* complex and *S. chamlongi* versus *S. nigrogilvum* and *S. tenobrosum* complex.

**Table 2:** Average wing centroid sizes (CS) in millimeters and statistical differences in wing size among seven human-biting black fly species

Species	*n*	Wing size (mm)		
		Mean ± SD	Minimum	Maximum
*S. asakoae*	50	2.64 ± 0.14^a^	2.21	2.91
*S. chamlongi*	30	3.03 ± 0.19^b^	2.62	3.31
*S. doipuiense* complex	39	2.72 ± 0.09^a^	2.56	2.98
*S. nodosum*	31	2.19 ± 0.12^c^	1.95	2.40
*S. nigrogilvum*	39	2.92 ± 0.14^b,d^	2.66	3.16
*S. tenebrosum* complex	31	3.17 ± 0.17^b,e^	2.86	3.75
*S. umphangense*	33	3.53 ± 0.15^f^	3.23	3.87

Different superscript letters denote statistically significant differences at *p* < 0.05

### Wing shape variation

**Fig. 4:**
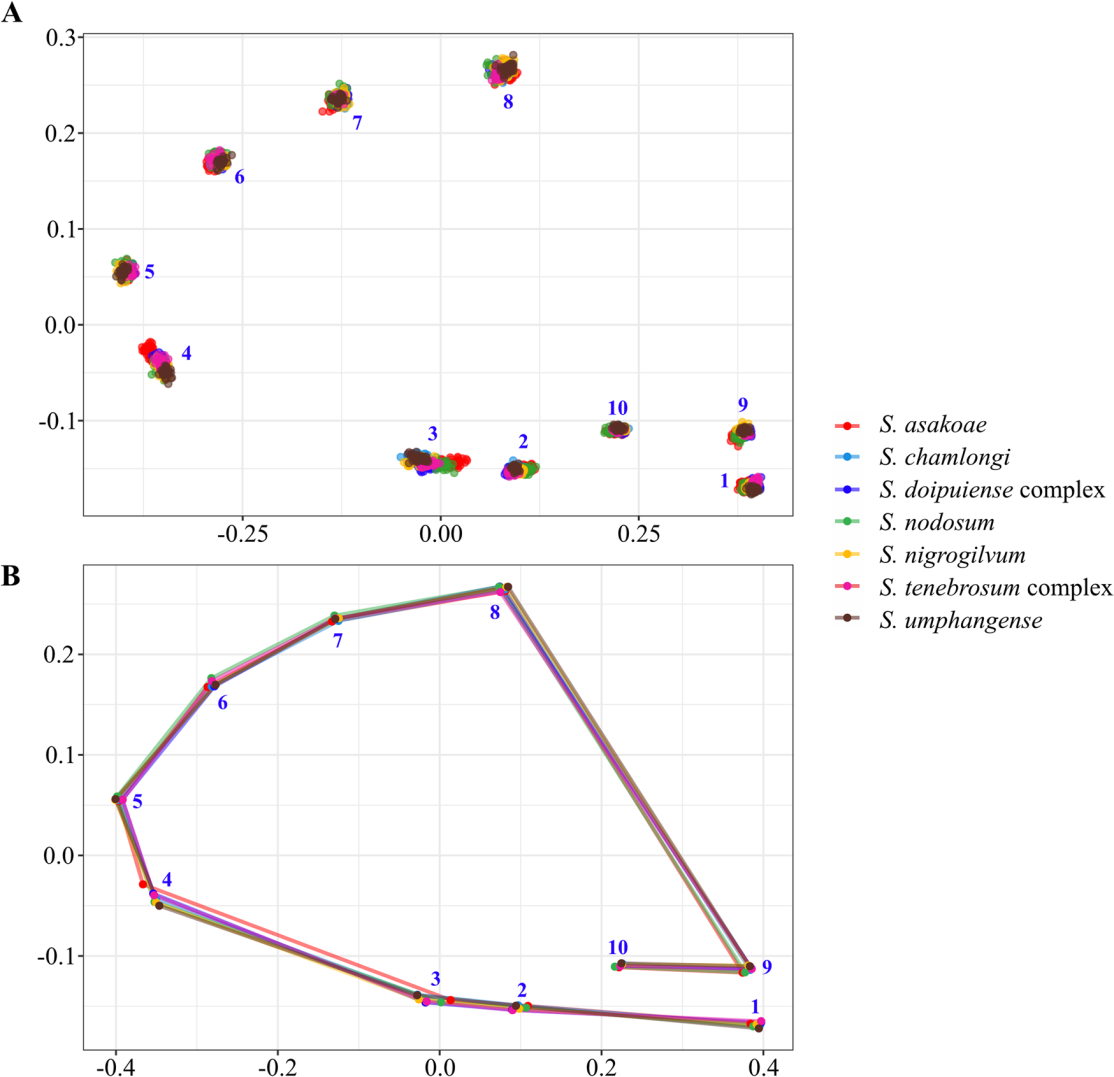
Shape differences in wing venation based on anatomical landmarks of seven black fly species. (**A**) Scatter plot showing residual coordinates of ten landmarks aligned by Procrustes analysis and (**B**) a wireframe graph showing the superposition of the overall mean shape

The visual comparisons of superimposition of the mean landmark configurations among seven black fly species showed the most noticeable landmark displacement in the lower part of the wing, particularly in landmark positions 3 and 4 (Fig. 4). The analysis also indicated that *S. asakoae* exhibited the most different wing shape, as its landmark position 4 clearly separated it from the other six species (Fig. 4).

**Fig. 5:**
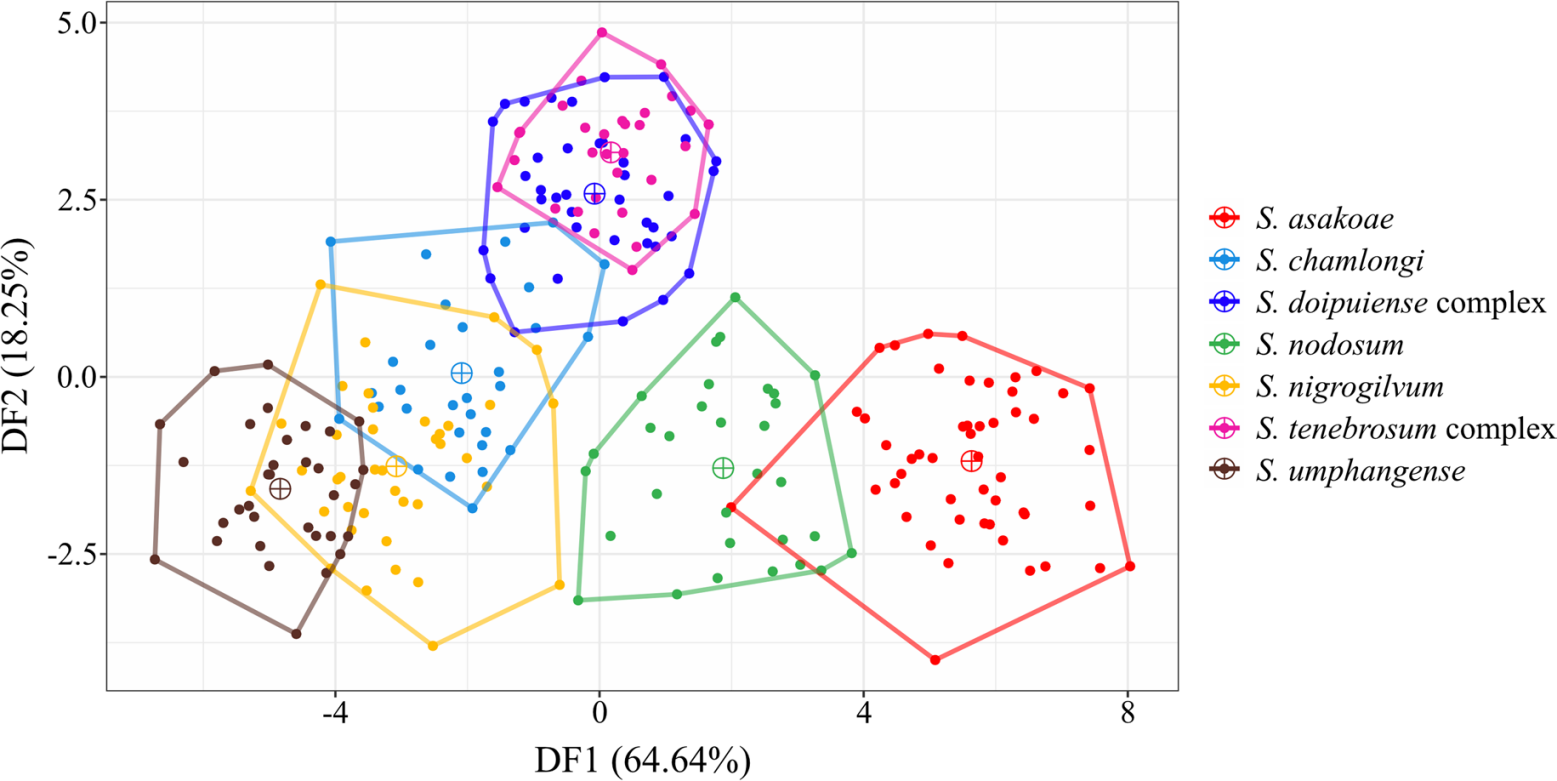
Factor map based on discriminant analysis (DA) showing the shape divergence of seven black fly species. Each polygon represents a different species, with dots indicating individual specimens and a sun cross marking the mean values for each species

Discriminant analysis of the wing shape variables among the seven black fly species showed clear separation, with a small overlap for two species, namely *S. asakoae* and *S. nodosum*. Conversely, the five other species revealed large overlapping among species, especially the two closely related species complexes, *S. tenebrosum* complex and *S. doipuiense* complex (Fig. 5). Despite the large overlap among species, significant differences (*p* < 0.05) in wing shape were observed among all black fly species based on the pairwise Mahalanobis distances (Table 3).

**Table 3:** Pairwise Mahalanobis distances and significant differences in wing shape of seven black fly species

Species	1	2	3	4	5	6	7
(1)* S. asakoae*	–						
(2) *S. chamlongi*	8.13^*^	–					
(3) *S. doipuiense* complex	7.03^*^	4.36^*^	–				
(4) *S. nodosum*	5.79^*^	6.13^*^	5.69^*^	–			
(5) *S. nigrogilvum*	8.86^*^	3.41^*^	5.13^*^	6.25^*^	–		
(6) *S. tenebrosum* complex	7.29^*^	4.77^*^	2.69^*^	5.65^*^	5.87^*^	–	
(7) *S. umphangense*	10.56^*^	4.24^*^	6.56^*^	7.68^*^	2.84^*^	7.11^*^	–

The superscript asterisks (*) after each Mahalanobis distance values denote statistically significant differences between black fly species at *p* < 0.05

### Phenetic relationships of wing shape among black fly species

**Fig. 6:**
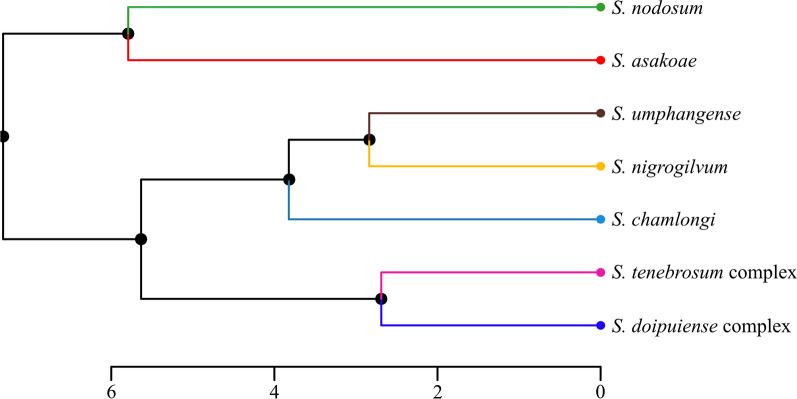
UPGMA dendrogram based on the Mahalanobis distances between average group shapes showing the phenetic relationship of wing shape among seven black fly species. The scale bar represents the Mahalanobis distance

The UPGMA dendrogram based on Mahalanobis distances demonstrated that the seven black fly species were separated into two distinct groups (Fig. 6). *Simulium nodosum* and *S. asakoae* formed one group, while the other five species formed another group, which was further divided into two subgroups: (1) *S. chamlongi* + (*S. umphangense* + *S. nigrogilvum*) and (2) *S. tenebrosum* complex + *S. doipuiense* complex.

### Validated classification

The success rate of landmark-based GM analysis of the wings for identifying seven black fly species is summarized in Tables 4 and 5. The size-based cross-validated classification revealed a low level of correct identification, with an overall accuracy score of 47.43% (120/253), while the shape-based cross-validated classification yielded a higher level of successful identification, with a total classification score of 88.54% (224/253). Furthermore, the shape-based classification showed a perfect result (100% accuracy score) for species identification of *S. asakoae*.

**Table 4:** Percentage of correct identification based on cross-validated classification of the wing size (centroid size) and wing shape (Mahalanobis distance values) of seven black fly species

Species	Percentage of correct classification (assigned/observed)	
	Based on wing size	Based on wing shape
*S. asakoae*	56.00% (28/50)	100.00% (50/50)
*S. chamlongi*	23.33% (7/30)	80.00% (24/30)
*S. doipuiense* complex	46.15% (18/39)	89.74% (35/39)
*S. nodosum*	29.03% (9/31)	96.77% (30/31)
*S. nigrogilvum*	41.03% (16/39)	76.92% (30/39)
*S. tenebrosum* complex	45.16% (14/31)	87.10% (27/31)
*S. umphangense*	84.85% (28/33)	84.85% (28/33)
Total performance	47.43% (120/253)	88.54% (224/253)

**Table 5:** Percentage of correctly assigned individuals based on the cross-validated classification of the wing shape of seven black fly species

Species	Classified as							Total	Correct identification
	1	2	3	4	5	6	7		
(1)* S. asakoae*	50	0	0	0	0	0	0	50	100.00%
(2) *S. chamlongi*	0	24	1	0	4	1	0	30	80.00%
(3) *S. doipuiense* complex	0	1	35	0	1	2	0	39	89.74%
(4) *S. nodosum*	0	1	0	30	0	0	0	31	96.77%
(5) *S. nigrogilvum*	0	4	1	0	30	0	4	39	76.92%
(6) *S. tenebrosum* complex	0	0	4	0	0	27	0	31	87.10%
(7) *S. umphangense*	0	0	0	0	5	0	28	33	84.85%
Correctly assigned/total individual								224/253	88.54%

The rows represent given species, while columns represent predicted species

### Sequence variation and genetic distance

**Fig. 7:**
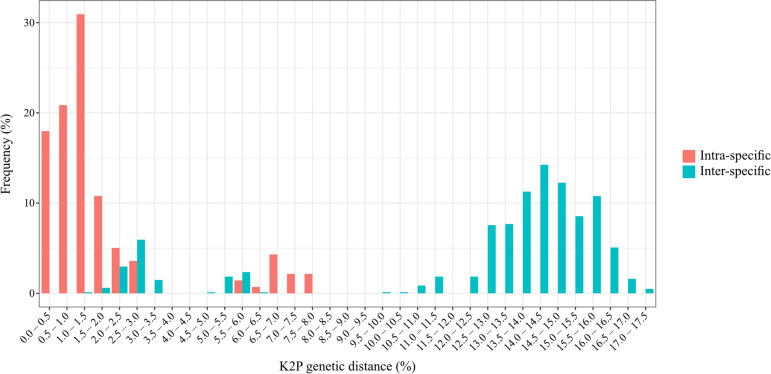
Frequency distribution of intraspecific and interspecific K2P genetic distances based on the *COI* gene of seven human-biting black fly species

In total, 70 *COI* sequences (658 bp long) of seven human-biting black fly species were obtained in this study, with 44 sequences identified as unique haplotypes. Sequence analysis revealed a high AT content, with an average base composition of A = 0.275, C = 0.178, G = 0.168, and T = 0.379. An overlap between the maximum intraspecific and minimum interspecific divergences was also noted, suggesting the absence of a barcoding gap (Fig. 7).

The maximum intraspecific genetic distance based on the K2P model ranged from 0.15% (*S. chamlongi*) to 7.92% (*S. asakoae*). Most species showed low intraspecific divergences (maximum value < 3%). The exception was *S. asakoae*, which displayed the highest intraspecific divergence (maximum value > 7%) (Table 6).

**Table 6:** Mean intraspecific (in bold) and interspecific genetic distances (%) with maximum values indicated in parentheses among seven human-biting black fly species

Species	1	2	3	4	5	6	7
(1)* S. asakoae*	**5.49 (7.92)**						
(2) *S. chamlongi*	15.69 (17.13)	**0.15 (0.15)**					
(3) *S. doipuiense* complex	14.97 (16.09)	13.01 (13.53)	**1.11 (1.86)**				
(4) *S. nodosum*	16.08 (17.00)	14.46 (14.79)	15.46 (16.14)	**0.23 (0.31)**			
(5) *S. nigrogilvum*	15.9 (17.07)	11.07 (11.38)	12.83 (13.75)	14.12 (14.42)	**0.73 (1.23)**		
(6) *S. tenebrosum* complex	14.91 (15.71)	13.22 (13.71)	2.64 (3.30)	14.61 (15.93)	13.21 (13.93)	**1.48 (2.81)**	
(7) *S. umphangense*	15.72 (16.48)	10.82 (11.24)	14.18 (14.89)	14.29 (14.67)	5.512 (6.06)	13.81 (14.11)	**0.58 (1.07)**

The mean interspecific genetic divergences ranged from 2.64% (*S. tenebrosum* complex versus *S. doipuiense* complex) to 16.08% (*S. asakoae* versus *S. nodosum*), with the highest value of 17.13% (*S. asakoae* versus *S. chamlongi*). Low interspecific genetic divergence values were observed in two species pairs: *S. tenebrosum* complex and *S. doipuiense* complex, and *S. nigrogilvum* and *S. umphangense*, with maximum values of 3.30% and 6.06%, respectively. Meanwhile the remaining species pairs showed very high levels of interspecific divergence, with maximum values exceeding 11% (Table 6).

### Species determination through a BLAST search

Using a BLAST search, all haplotypes could be identified to the species level (Table S1). Four out of seven species, including *S. nodosum*, *S. tenebrosum* complex, *S. nigrogilvum*, and *S. umphangense* were correctly classified to their respective species or complex with high sequence similarity (98–100%). On the other hand, seven haplotypes of *S. asakoae* displayed the highest similarity (> 99%) with several different species of the *S. asakoae* species-group as follows: *S. nanthaburiense* (H1), *S. chaowaense* (H4), *S. pitasawatae* (H5), *S. tamdaoense* (H6), and *S. asakoae* (H3, H7), whereby the H2 haplotype was identical to three different species, namely *S. asakoae*, *S. vinhphucense*, and *S. hongthaii*. *S. chamlongi* and *S. doipuiense* complex were also ambiguously assigned to the species level. One haplotype (H9) of *S. chamlongi* matched perfectly to *S. phuluense*, while the other (H8) was identical to both *S. phuluense* and *S. chamlongi*. Additionally, the majority of haplotypes (9/10) of the *S. doipuiense* complex showed the highest similarity (> 98%) to the *S. tenebrosum* complex, with only one (H14) being accurately identified as the *S. doipuiense* complex with a sequence similarity of 98.63%.

### Efficiency of the *COI* gene for species identification

Species identification based on the *COI* sequences demonstrated high success rate, with 98.57% (69/70) correct identification for both the BM and BCM methods. The only exception was the misidentification of one specimen (TN5) of *S. tenebrosum* complex as *S. doipuiense* complex.

### Phylogenetic analysis

**Fig. 8:**
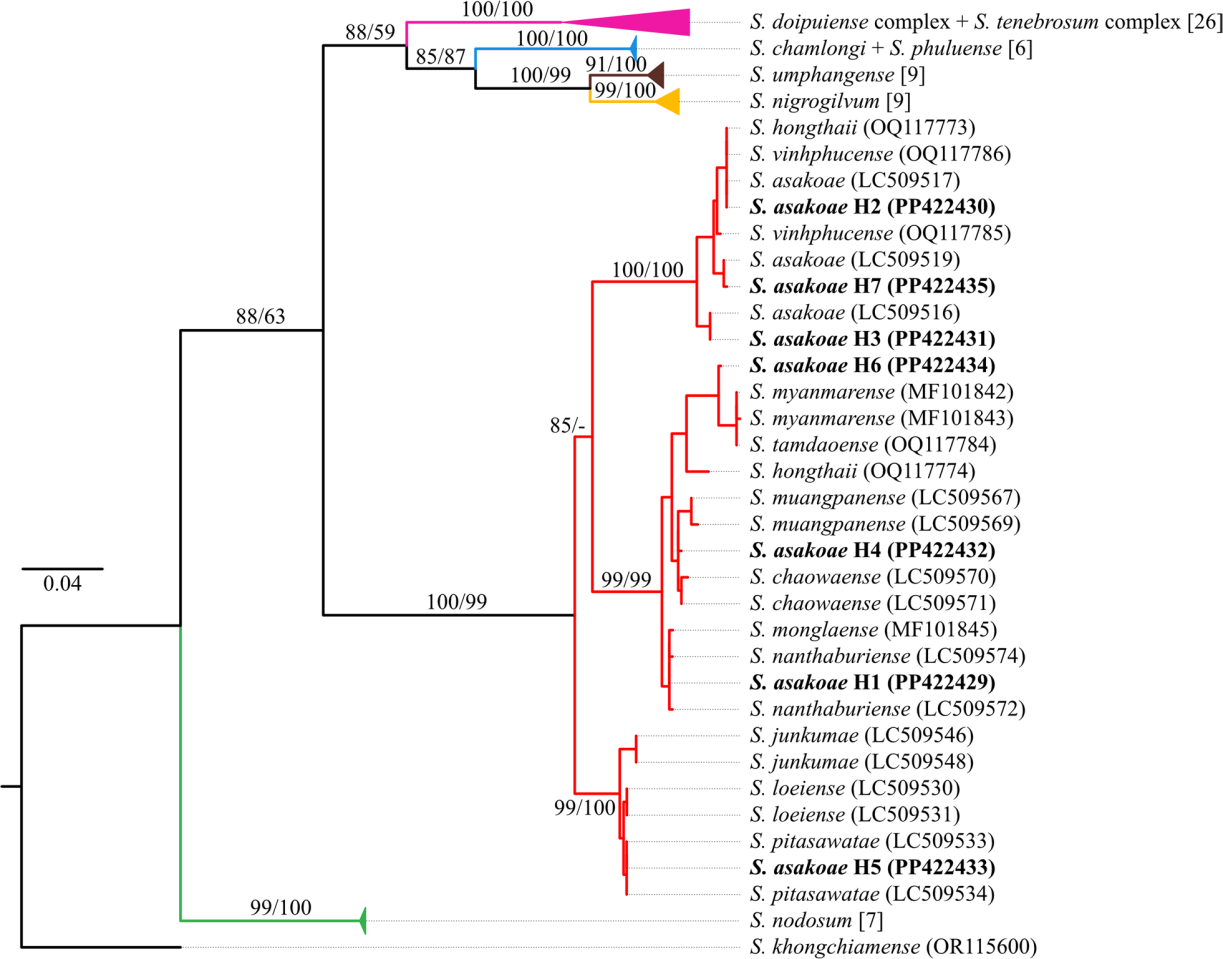
Maximum likelihood phylogenetic tree based on 585 bp *COI* gene of seven human-biting black fly species and their related species. Bootstrap support values (ML/NJ) greater than 50% are indicated near the branches. Some distinct clades were collapsed for clearer presentation, and the number of sequences falling within those clades is indicated in square brackets. Sequences obtained in this study are highlighted in bold type. All sequences used for constructing the tree are detailed in Table S2

Both NJ and ML phylogenetic trees based on the *COI* sequences yielded similar tree topologies. Thus, only the ML tree is demonstrated (Fig. 8). Three species, namely *S. nigrogilvum*, *S. nodosum*, and *S. umphangense* were identified as distinct species, forming their own monophyletic groups with strong bootstrap support. In contrast, the remaining four species—*S. asakoae*, *S. tenebrosum* complex, *S. doipuiense* complex, and *S. chamlongi*—were not monophyletic. Sequences of *S. chamlongi* were clustered with *S. phuluense* from Vietnam, while the sequences of *S. tenebrosum* complex were intermixed with *S. doipuiense* complex. Notably, seven haplotypes of *S. asakoae* were divided into three subclades and placed into different species members of the *S. asakoae* species-group as follows: (1) *S. asakoae*/*S. vinhphucense*/*S. hongthaii* for H2, H3, and H7; (2) *S. pitasawatae* for H5; (3) *S. tamdaoense*/*S. myanmarense* for H6; (4) *S. nanthaburiense*/*S. monglaense* for H1; (5) *S. chaowaense* for H4.

### Species delimitation analysis

**Fig. 9:**
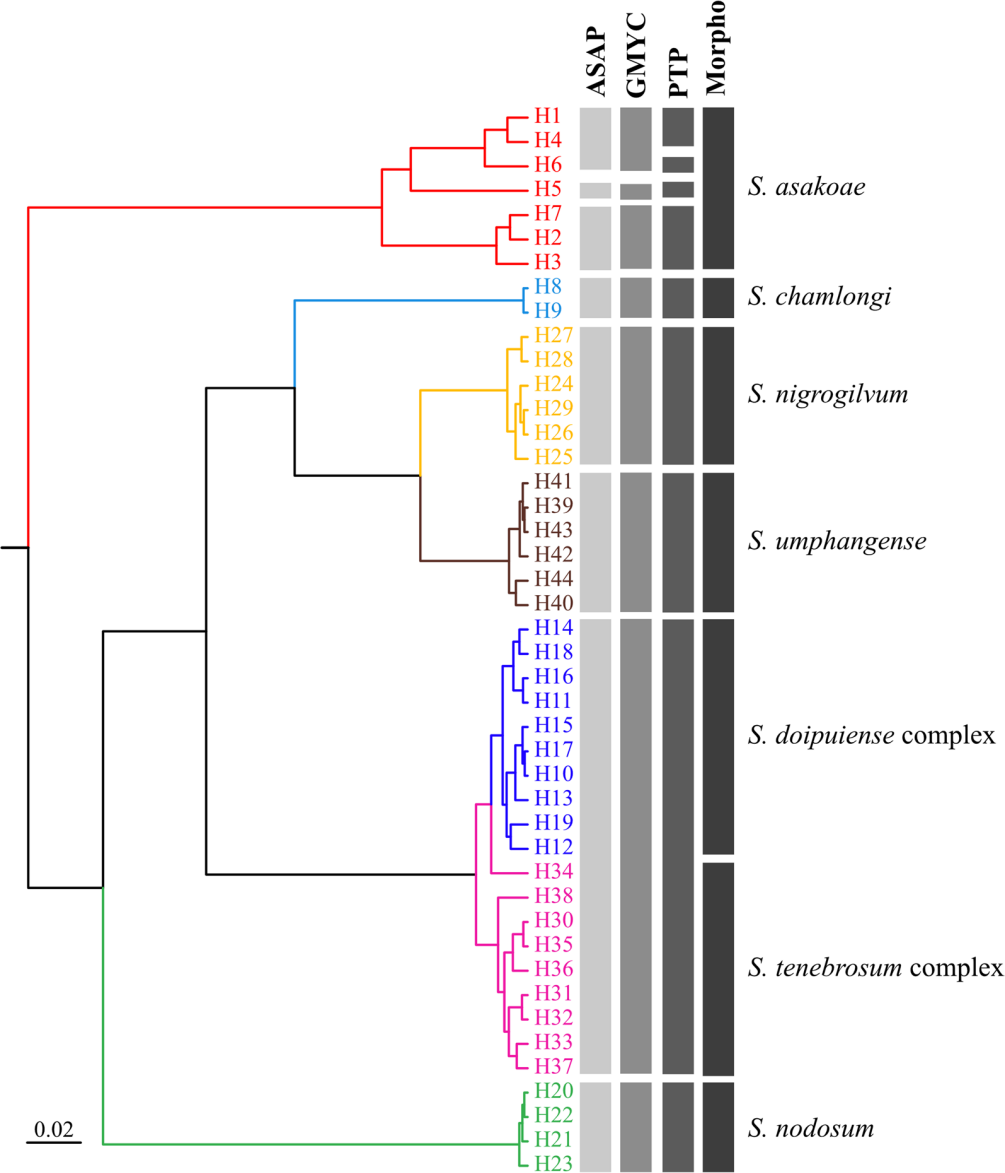
Summary of the three species delimitation analyses (ASAP, GMYC, and PTP) based on the *COI* haplotypes from seven different nominal species of black flies. The maximum clade credibility tree built from BEAST is colored according to morphospecies. The vertical bars at the tips of the tree correspond to the result of each species delimitation method and morphological identification, respectively. Detailed information of each haplotype is provided in Table S2

Three species delimitation methods (ASAP, PTP, and GMYC) mostly supported seven morphospecies, as summarized in Fig. 9. Both ASAP and PTP methods identified eight putative species, while GMYC method recovered more putative species (nine species). All species delimitation methods merged *S. tenebrosum* complex and *S. doipuiense* complex into a single species, disagreeing with their morphologically defined species. Regarding *S. asakoae*, seven haplotypes were divided into three (ASAP and PTP) up to four (GMYC) putative species, suggesting cryptic diversity in this species.

## Discussion

Here, we demonstrate for the first time that landmark-based GM of wing shape is a promising tool for the identification of the adult human-biting black flies in Thailand on a species level, although not with perfection. As expected, wing shape analysis is superior to wing size analysis in distinguishing black fly species, consistent with findings in numerous previous reports across various insect groups [46–48, 89, 90]. Unlike wing size, which is susceptible to environmental influences and is not a suitable parameter for distinguishing species [57, 91], wing shape appears to be a fixed trait that preserves genetic information, making it a desirable species-specific indicator [92, 93]. Our allometric analysis also indicated that one variable (wing size) tends to increase, while the other (wing shape) tends to decrease (shows a negative correlation), as has been observed in several medically and forensically important insects, e.g., *Anopheles barbirostris* mosquitoes [46], *Armigeres* mosquitoes [50], *Culex vishnui* subgroup [94], and *Stomoxys* flies [90]. Therefore, this study excluded wing size data before wing shape analysis. Noticeably, our GM analysis based on wing shape was often unable to correctly assign the closely related species to their respective species. This result aligns with expectations, given the high morphological similarities between *S. umphangense* and *S. nigrogilvum*, where species separation relies mainly on subtle difference in the leg color of the fore tibia [14]. Similarly, the distinction of the two closely related species complexes, *S. tenebrosum* complex and *S. doipuiense* complex, is feasible based on certain features of the legs, specifically the coloration of the hind tibia and basitarsus [95].

The phenotypic relationships among seven black fly species mostly align with the phylogenetic tree based on the *COI* gene, suggesting that landmark-based GM analysis of wing shape could be used as an alternative tool for evolutionary studies, taxonomy, and systematics [51, 61, 94]. However, both phenotypic and phylogenetic analyses erroneously placed *S. nodosum* in the wrong group, contradicting its morphological classification and previous molecular studies [58, 96]. Instead of assigning all seven species to their respective subgenus, *S. nodosum* of the subgenus *Simulium* s.l. was placed in a distinct group or clustered as a sister species with *S. asakoae* of the subgenus *Gomphostilbia*. The incorrect assignment of *S. nodosum* seems to be due to the lack of intermediate taxa in the analyses. Therefore, incorporating additional black fly specimens of different species and subgenera into the analyses is recommended to improve both phenotypic and phylogenetic accuracy [97, 98].

In this study, we also assessed the performance of wing GM analysis in comparison to DNA barcoding based on the *COI* gene for distinguishing morphologically defined species. DNA barcoding clearly outperformed wing GM analysis, demonstrating an almost perfect efficiency (> 98%) in species identification using both BM and BCM methods. The high accuracy (> 90%) of DNA barcoding in distinguishing Thai black fly species was also observed in previous studies, even when analyzing a larger number of species (41–89 nominal species) [35, 96]. Further, our phylogenetic analysis of the *COI* gene coupled with species delimitation analysis revealed hidden diversity within *S. asakoae*, suggesting the presence of up to four putative species. A previous molecular study using the *COI* gene also indicated that *S. asakoae* in Thailand consisted of at least seven groups [34]. Subsequent investigations, which extensively analyzed a large number of morphologically defined *S. asakoae* specimens further confirmed this observation, with a total of 23 new species described in recent years [99–101]. The high morphological variability and the high similarity of the *COI* sequences of several members of the *S. asakoae* species-group hampers the correct assignment of our *S. asakoae* specimens to the defined morphospecies [99, 102]. Initially, DNA barcoding was considered highly effective in distinguishing the true *S. asakoae* from other members in the *S. asakoae* species-group [102]. However, a recent study in Vietnam [39], as well as this study, demonstrated that the *COI* gene is no longer a suitable barcoding marker. In these studies, two related species from Vietnam, namely *S. hongthaii* and *S. vinhphucense*, were placed in the same clade as the true *S. asakoae*, leading to their delimitation as a single species. Some previous reports also suggested that the *COI* gene, or even the rapidly evolving nuclear *BZF* gene provide insufficient signal to distinguish most members of the *S. asakoae* species-group [35, 39, 99, 103]. To overcome this problem, further studies using more variable gene markers, such as the elongation complex protein 1 (*ECP1*), the 5-intron gene (*5intG*) [104], or molybdenum cofactor sulfurase (*MCS*) [105], are required to enhance species differentiation within the *S. asakoae* species-group. Additionally, we found that DNA barcoding was ineffective in identifying *S. tenebrosum* complex and *S. doipuiense* complex, and three species delimitation methods further supported this result by merging them as a single species [35, 39]. A more recent study examining the efficiency of two rapidly evolving nuclear genes suggested that the *ECP1* gene is a promising barcoding marker for the successful identification of the two species complexes [106]. The molecular analysis in this study also revealed a similar result that *S. chamlongi* is not monophyletic as its clade including *S. phuluense* from Vietnam [39]. It may be presumed that *S. chamlongi* and *S. phuluense* are morphologically distinct but molecularly homosequential, since they are readily distinguished by the color of female legs, the number of male upper-eye (large) facets and the presence or absence of tubercles on the pupal head [107, 108. This suggests the need for additional genetic markers beyond the *COI* gene to resolve their phylogenetic relationships. 

Although the accuracy of landmark-based GM of wing shape for black fly species identification in this study does not reach that of DNA barcoding, the results suggest that it is a promising complementary method to traditional and molecular methods. In addition, wing GM analysis is simple, reliable, cost-effective, rapid, and only needs nondamaged wings, in contrast to molecular identification [47, 61, 109]. Since this is the first study that applies wing GM analysis for black fly species identification, we focus on the most important species (anthropophilic species) and use the most popular method, landmark-based GM [56, 57]. Further studies that include more species of different subgenera and employ both landmark- and outline-based GM approaches are needed to determine the extent to which wing GM analysis can serve as a tool for identifying adults of black fly species.

## Conclusions

In this research, we demonstrate that landmark-based GM analysis of wing shape, which achieves greater accuracy than wing size analysis in species identification, is a promising, reliable tool for supplementing and enhancing morphological identification of black fly adults. Our molecular analysis suggests that there may be up to four putative species within the morphologically defined *S. asakoae*.

## Supplementary Information

Additional file 1: Table S1. Results from the NCBI BLAST search of black fly samples based on *COI* gene

Additional file 2: Table S2. Details of *COI* sequences generated from the present study and retrieved from NCBI GenBank databased used for molecular analysis

## Data Availability

The authors confirm that all data supporting the findings of this study are available within the article. All sequences generated from the study have been deposited in the GenBank database with assigned accession numbers PP422429–PP422472.

## Abbreviations

ASAP: Assemble species by automatic partitioning
BCM: Best close match
BM: Best match
COI: Cytochrome *c* oxidase subunit I
GM: Geometric morphometrics
GMYC: Generalized mixed yule coalescent
PCR: Polymerase chain reaction
PTP: Poisson tree processes
